# PSP‐Richardson's Syndrome as a Rare Phenotypic Expression of Very Late‐Onset Huntington's Disease: A Case Report

**DOI:** 10.1002/mdc3.13943

**Published:** 2024-01-03

**Authors:** Stephane Prange, Chloé Laurencin, Pauline Roche, Isabelle Quadrio, Stéphane Thobois

**Affiliations:** ^1^ Hospices Civils de Lyon, Department of Neurology C, Expert Parkinson Center NS‐PARK/FCRIN Pierre Wertheimer Neurological Hospital Bron France; ^2^ Univ Lyon, Marc Jeannerod Cognitive Neuroscience Institute, CNRS, UMR 5229 Bron France; ^3^ Univ Lyon, Faculté de Médecine et de Maïeutique Lyon Sud Charles Mérieux Université Claude Bernard Lyon 1 Oullins France; ^4^ Hospices Civils de Lyon, Neurobiology and Neurogenetics Department of Biochemistry and Molecular Biology Lyon France

**Keywords:** Huntington's disease, progressive supranuclear palsy, Richardson's syndrome

Huntington's disease (HD) is an autosomal dominantly inherited neurodegenerative disease due to increased (≥36) CAG repeats in the first exon of the huntingtin gene (HTT). HD is classically characterized by generalized chorea, and slowly progressive parkinsonism and dystonia associated with psychiatric and cognitive disorders.^1^ However, very unusual presentations may also exist such as the one reported here.

A 79‐year‐old man was first referred to our movement disorder unit for gait disorders, imbalance, and frequent falls lasting two years. He had no personal medical history and did not take any medication. Importantly, his older brother presented with mild chorea, had marked striatal atrophy on brain MRI, and was diagnosed with HD (40 ± 1 CAG repeats, with 17 repeats on the other allele) at the age of 81. The mother developed movement disorders at the age of 75 and died at 81 without genetic testing. No other of the eight siblings and their offspring had known neurological disorders. The patient had no children.

At that time, the patient had slow gait with reduced bilateral arm swing and bradykinesia, flexed posture, postural instability with frequent falls, hypomimia, slurred speech, and slowness of vertical saccades. The patient reported no recent behavioral or mood changes. Two years later, he was bedridden, with severe bilateral, akinetic‐rigid parkinsonism, left hand dystonia, and dysarthria (Video 1, Segment 1). He had slow and hypometric horizontal saccades, with more severe impairment of vertical saccades and pursuit, but preserved oculocephalic reflex (Video 1, Segment 2). No chorea was observed at any time, in contrast to his older brother who had permanent chorea since the onset, which deteriorated after the withdrawal of tetrabenazine. He was demented with a dysexecutive syndrome but preserved long‐term memory (MoCA 16/30; FAB 14/18). Altogether, phenotypic presentation and rapid progression led us to diagnose progressive supranuclear palsy‐Richardson's Syndrome (PSP‐RS). Brain MRI was in favor, showing mesencephalic atrophy with a hummingbird sign and bilateral frontal atrophy, without marked striatal atrophy (Fig. 1A–C). Levodopa treatment up to 1000 mg daily was tried for 12 months without any success. Due to positive family history, molecular testing of HD was performed and revealed pathological HTT expansion with 40 ± 1 CAG repeats (19 repeats on the other allele).

**Video 1:** The video shows an 81‐year‐old man with very late‐onset Huntington's disease manifesting as progressive supranuclear palsy‐Richardson's syndrome, with major bradykinesia, dysarthria, amimia and supranuclear gaze palsy with preserved oculocephalic reflex. The patient is bedridden and unable to walk or stand‐up.

**Figure 1:**
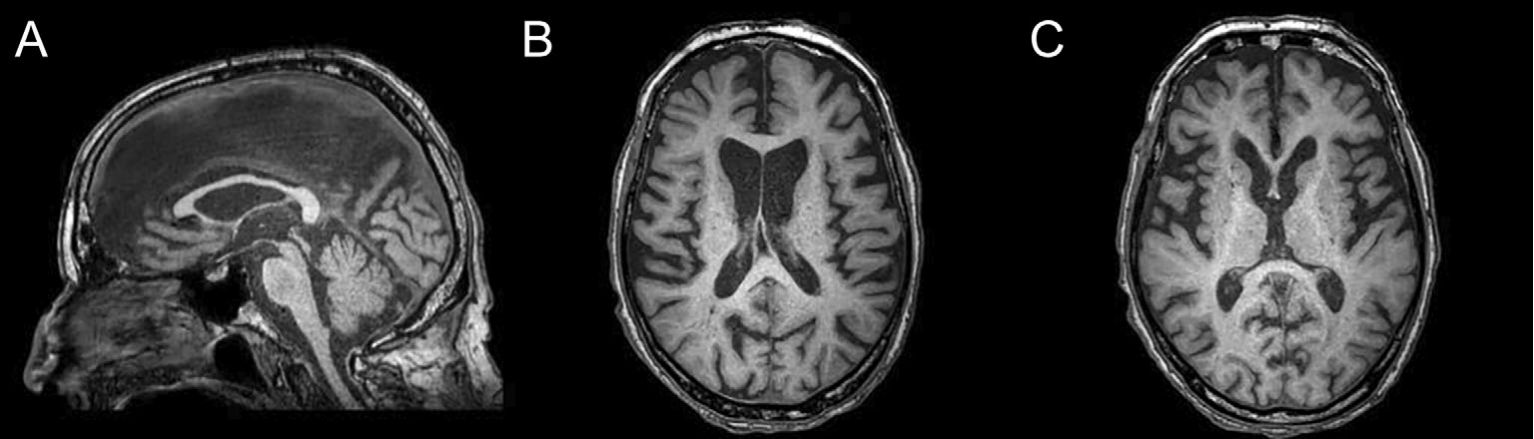
Brain MRI. Sagittal T1‐weighted image (**A**) showing midbrain atrophy with preserved pons corresponding to the hummingbird sign. Pons to midbrain area ratio (P/M = 6.01), third ventricle to frontal horns width ratio (3rdV/FH = 0.29) and Magnetic Resonance Parkinsonism Index 2.0 (MRPI 2.0 = 3.89) index indicated prominent midbrain atrophy, as observed in PSP‐RS. Axial T1‐weighted images showing bilateral frontal atrophy (**B**) without caudate atrophy (**C**).

This case illustrates a very uncommon clinical presentation corresponding to PSP‐Richardson's syndrome with severe and rapidly progressive parkinsonism without chorea in a 79‐year‐old patient with fully penetrant, pathogenic HTT expansion (≥40 CAG repeats) and family history of very late‐onset choreic HD. Thereby, this remarkable case raises several hypotheses regarding the phenotypic expression of these rare degenerative disorders in a family with late‐onset HD. First, we may consider that rare atypical parkinsonism may incidentally co‐occur in a patient with HD and that severe parkinsonism as observed in PSP‐RS may supersede chorea, although the probability is extremely low given the low prevalence rates of both diseases.^1^, ^2^ Second, we cannot exclude that the patient has PSP‐RS but remains presymptomatic for HD after age 80. Indeed, low‐repeat HTT expansions are characterized by later onset of HD, but also greater variance (11.8 years). Overall, CAG‐repeat length explains up to 65% of the variance,^3^, ^4^ which is further modified by the length of uninterrupted CAG repeats (not tested for our patient)^3^ and several DNA maintenance genes.^1^ However, the likelihood of such hypothesis remains low and CAG repeats of 40 and more are considered fully penetrant, with mean predicted motor onset at 62.6 years.^5^

Third, we postulate that very late‐onset HD may manifest as PSP‐RS in line with recent reports. Indeed, apart from the classical choreic phenotype, HD may manifest as prominent akineto‐rigid parkinsonism without chorea in young patients with large CAG expansion (Westphal variant).^6^ In addition, adult‐onset HD patients with mild or no chorea but prominent parkinsonism have greater cognitive and neuropsychiatric disturbances,^7^ some with dysautonomia^8^ or oculomotor abnormalities.^9^ Notably, oculomotor abnormalities are frequent in HD, particularly for voluntary saccades, with impaired initiation including in presymptomatic patients.^10^ However, slowing of both horizontal and vertical saccades is more prominent in advanced HD and in patients with Westphal syndrome, pinpointing the role of brainstem pathology,^10^ as observed in advanced PSP‐RS. Fully penetrant pathogenic HTT expansions were also demonstrated in patients with progressive supranuclear palsy/frontotemporal dementia and amyotrophic lateral sclerosis, without chorea.^2^ Recently, PSP‐RS phenotype was reported in a 58‐year‐old male patient with 41 CAG repeats and familial history of choreic HD.^11^ Remarkably, our patient had 40 CAG repeats, as did his older brother with choreic HD of maternal inheritance, corresponding to fully penetrant alleles.

The factors which modify HD phenotype in carriers of pathogenic alleles are yet to be elucidated. Interestingly, variable phenotypic expression of HD was also observed in monozygotic twins.^12^ One hypothesis is that clinical presentation might be related to regional and cell‐specific CAG instability in brain structures, which may underpin the variable patterns of cortical and subcortical atrophy and be possibly favored by several genetic modifiers such as the length of uninterrupted CAG‐repeats.^3^ Indeed, increased somatic instability in the motor and frontal cortex was observed in patients with frontotemporal dementia/amyotrophic lateral sclerosis without chorea who carried pathogenic HTT repeat expansion and had cortical huntingtin deposits, whereas no striatal degeneration was observed.^2^ Moreover, greater somatic CAG instability in the pons, thalamus and amygdala was observed in a 73‐year‐old patient with PSP phenotype and pathological HTT expansion, while the caudate was less affected.^13^ Consistent with this observation, it is noteworthy that no major caudate atrophy was observed in our patient, whereas mesencephalic atrophy was prominent. Interestingly, the prevalence of patients with low penetrance alleles remains low (<0.3%) in cohorts of adults with frontotemporal dementia/amyotrophic lateral sclerosis, Lewy body dementia,^2^ and Parkinson's disease,^14^ whereas intermediate (27 to 35 repeats) alleles carriers are more frequent in patients with definite MSA, some of them positive for polyglutamine staining in the pons and basal ganglia.^14^ Notably, polyglutamine deposits may be present in association with α‐synuclein, TDP‐43 or tau aggregates in patients with pathogenic HTT CAG repeats,^1^, ^2^, ^13^ pinpointing the complex interaction of these protein aggregates. All these observations might be confirmed in future pathological studies.

Altogether, the present case emphasizes that genetic testing of HD should be considered in patients with atypical parkinsonism, even in the absence of chorea, in case of positive family history of HD. This raises the awareness that very late‐onset HD with low CAG repeats possibly manifests as atypical, non‐choreic, phenotypes, which questions the role of somatic instability and differential atrophy of the striatum versus the brainstem in typical versus atypical HD.

## Author Roles

(1) Research project: A. Conception, B. Organization, C. Execution; (2) Statistical Analysis: A. Design, B. Execution, C. Review and Critique; (3) Manuscript Preparation: A. Writing of the first draft, B. Review and Critique.

S.P.: 1A, 1B, 1C, 2B, 3A

C.L.: 1C; 3B

P.R.: 1C, 3B

I.Q.: 1B, 1C, 3B

S.T.: 1A, 1B, 1C, 2A, 3A

## Disclosures

**Ethical Compliance Statement:** This study was approved by the institutional Scientific and Ethics Committee (Date 21 March 2023, No. 23_103). The patient signed written informed consent regarding publishing their data and photographs. All authors confirm that they have read the Journal's position on issues involved in ethical publication and affirm that this work is consistent with those guidelines.

**Funding Sources and Conflicts of Interest:** No specific funding was received for this work. The authors declare that there are no conflicts of interest relevant to this work.

**Financial Disclosures for the Previous 12 Months:** The authors declare that there are no additional disclosures to report.
